# Comparison of patient outcomes between video and non-video laryngeal mask airway insertion performed by novices: a prospective randomized controlled study

**DOI:** 10.3389/fmed.2025.1667040

**Published:** 2025-10-30

**Authors:** Ye Jiang, Fang Xing, Qian Wu, Yanan Gao, Guozhong Chen, Cheng Li

**Affiliations:** Department of Anesthesiology and Perioperative Medicine, Shanghai Key Laboratory of Anesthesiology and Brain Functional Modulation, Clinical Research Center for Anesthesiology and Perioperative Medicine, Translational Research Institute of Brain and Brain-Like Intelligence, Shanghai Fourth People's Hospital, School of Medicine, Tongji University, Shanghai, China

**Keywords:** laryngeal mask airway, visual intubation laryngeal mask airway, learning effect, novice, placement accuracy

## Abstract

**Objective:**

This study compares the patient outcomes of video laryngeal mask airway (V-LMA) and non-video laryngeal mask airway (NV-LMA) to assess which is easier for novices to master, achieves faster placement success, and causes least injury to the patients.

**Methods:**

Twenty novice practitioners (resident doctors/anesthesia nurses) from the Department of Anesthesia and Perioperative Medicine, Shanghai Fourth Peoples' Hospital, were randomized 1:1 to the V-LMA or NV-LMA group. After standardized training, the participants performed supervised LMA insertions on 60 patients. The learning outcomes and patient injury rates during LMA placement were compared between the groups.

**Results:**

Both groups achieved 100% first-attempt success. The V-LMA group demonstrated superior bronchoscope alignment (90% vs. 50%, *P* = 0.001). Postoperative throat pain was experienced by patients in both groups, but the V-LMA group demonstrated a lower 1-h incidence of postoperative throat pain (20% vs. 46.7%, *P* = 0.028), with better intraoperative hemodynamic stability.

**Conclusion:**

When inserted by novice practitioners, the V-LMA improves placement accuracy and reduces patient injury compared with the NV-LMA.

**Clinical trial registration:**

ChiCTR2300069399.

## 1 Introduction

Since its invention in 1983, the laryngeal mask airway (LMA) has become a cornerstone in anesthesia practice (1, 2). As a supraglottic airway device, the LMA enables ventilation without tracheal intubation (3), and it is now recommended in standard practice guidelines for routine airway management (4). The LMA is widely used in clinical anesthesia, emergency care, and intensive care unit settings (5).

Compared with tracheal intubation, LMA insertion is simpler, faster to learn, and associated with fewer complications (6–8). According to the American Society of Anesthesiologists (ASA) guidelines, LMA insertion has become an important tool for managing difficult ventilation (9). Studies have indicated that when laryngoscope intubation fails, LMA insertion can be performed to restore ventilation and maintain oxygenation (10, 11).

However, traditional LMAs are unreliable. For instance, challenges like mispositioning due to body movement, as well as air leakage (usually indicated by indirect evidence, such as changes in tidal volume or an audible gas leak) and other complications, have been reported (12). Severe displacement increases the risk of gastric reflux, aspiration, and trauma (13–15); therefore, displacement should be avoided.

Recently, video LMAs (V-LMAs) have been introduced, which provide real-time feedback during LMA insertion, allowing immediate positional adjustments (16). However, it remains to be clarified whether there is a difference in the placement success rate between traditional non-video LMAs (NV-LMAs) and V-LMAs. Broader adoption of V-LMAs could expand their use beyond anesthesiologists, improving timely ventilation in critical scenarios.

This study was designed to compare V-LMAs and NV-LMAs to determine which enables faster mastery by novices, has higher placement success, and causes least patient injury.

## 2 Methods and materials

### 2.1 Study design and participants

Twenty novice practitioners (resident doctors/anesthesia nurses) from the Department of Anesthesia and Perioperative Medicine, Shanghai Fourth People's Hospital, were recruited for this study. The study was approved by the institutional ethics committee (approval number 2022165-001) and has been registered in the Chinese Clinical Trial Registry (ChiCTR2300069399; 15 March 2023). Informed consent was obtained from all patients.

All practitioners had no prior LMA insertion experience. The practitioners were randomized 1:1 to two groups: (1) the V-LMA group and (2) the NV-LMA group, using a random number table. Sixty patients were also randomized 1:1 to the V-LMA group and the NV-LMA group using a random number table through the allocation manager, who stored the randomization table. The patients were informed preoperatively that they would be randomly assigned to either group, but they were blinded to the group allocation. The independent statisticians were also blinded, solely analyzing LMA efficacy without knowing any allocation information. The randomization numbers were only provided to a specific nurse who was not involved in the data analysis. Therefore, the grouping information was known only by the allocation manager, the specific nurse, anesthesiologists, surgeons, and data collectors.

### 2.2 Inclusion and exclusion criteria

The patient inclusion criteria were (1) ASA physical status classification I–II; (2) aged 18–70 years; (3) cardiac function class I–II; (4) body mass index (BMI) 18–25 kg/m^2^; (5) surgical duration < 3 h; and (6) voluntary provision of informed consent. The patient exclusion criteria were (1) abnormal airway anatomy (mouth opening < 3.0 cm, thyromental distance < 6.5 cm, micrognathia, Mallampati classification ≥3); (2) maxillofacial surgery, prone/beach chair position, or one-lung ventilation; (3) high reflux and aspiration risk; (4) throat pain or discomfort; (5) obstructive lung disease (asthma, chronic obstructive pulmonary disease, etc.); and (6) significant dental abnormalities (loose teeth, severe misalignment).

### 2.3 Research procedures

Under the supervision of the senior anesthesiologist, the practitioners in the V-LMA group received training on visual insertion techniques, while the practitioners in the NV-LMA group learned regular intubation laryngeal mask insertion techniques.

**Figure 1:**
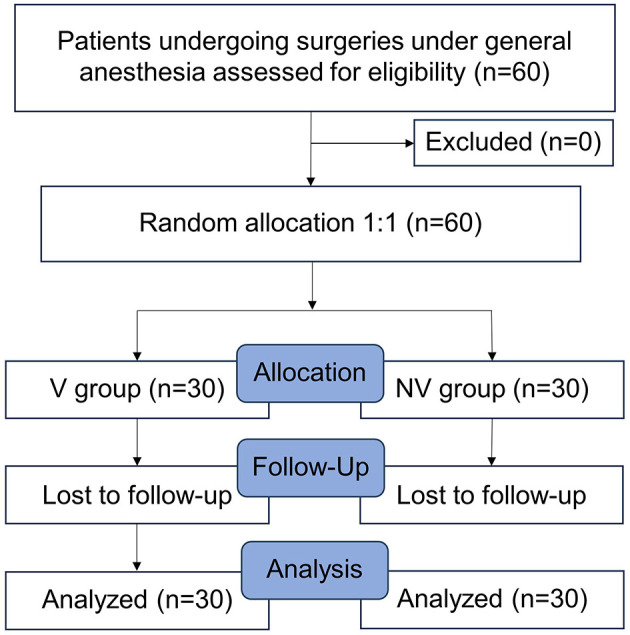
Flow diagram.

After the training, the practitioners performed supervised insertions on elective surgery patients under general anesthesia. The learning outcomes and patient injuries during insertion were analyzed. The study flow diagram is summarized in Figure 1.

#### 2.3.1 Preparation before clinical practice

Novice practitioners (anesthesia doctors/nurses with no prior LMA insertion experience) underwent structured preparation by studying glottic anatomy and insertion protocols; reviewing instructional videos; observing mentors perform three supervised insertions; and practicing on simulation mannequins until achieving three consecutive successful insertions. The mentors were senior anesthesiologists with ≥5 years of LMA insertion expertise.

#### 2.3.2 Clinical practice implementation

Each practitioner performed laryngeal mask insertion on three patients randomly in each group. Prior to the induction of anesthesia, all patients underwent standard monitoring (electrocardiogram, pulse oximetry, and non-invasive blood pressure monitoring every 3 min [every 1 min during anesthetic induction]). Pre-oxygenation with 100% oxygen (5 L/min) was performed for 5 min. The induction agents included intravenous propofol (1.50 mg/kg), sufentanil (0.40 μg/kg), and rocuronium (0.6 mg/kg). The LMA was inserted 5 min after the induction of anesthesia using a single-person technique and inflated to 50 cmH_2_O using a pressure gauge. Ventilation was initiated after confirming bilateral breath symmetry, two consecutive partial pressure of end-tidal carbon dioxide (PETCO_2_) waveforms, and the absence of oropharyngeal leakage. The mechanical ventilation parameters included oxygen flow at 2.0 L/min, tidal volume of 7 mL/kg, and respiratory rate of 12 breaths/min. Successful insertion required bilateral chest excursion, clear lung sounds, PETCO_2_ waveform, and no leakage. Failed insertion after three attempts prompted endotracheal intubation. Maintenance of anesthesia was achieved using propofol (4–12 mg/kg/h) and remifentanil (0.05–2.00 μg/kg/min) until completion of surgery. Fiberoptic bronchoscopy grade was defined as follows (17, 18): Grade 1: visualization of the glottis; Grade 2: visualization of the glottis and the lingual surface of the epiglottis; Grade 3: visualization of the glottis and the laryngeal surface of the epiglottis; Grade 4: no glottis visible. Data collection commenced from this point.

#### 2.3.3 Laryngeal mask size selection

The SaCo VLM (Zhejiang U-Yue Medical Equipment Co. Ltd.) was used for V-LMA, while the Proseal Laryngeal Mask (Henan Tuoren Medical Equipment Co. Ltd.) was used for NV-LMA. The mask size was determined based on the patient's weight, as follows: Size 3: 30–50 kg; Size 4: 50–70 kg; Size 5: ≥70 kg.

### 2.4 Outcomes

#### 2.4.1 Primary outcome

The primary outcome was the first-attempt insertion success rate.

#### 2.4.2 Secondary outcomes

The secondary outcomes were the (1) time to successful insertion; (2) fiberoptic bronchoscopy grade (19); (3) frequency of *in situ* adjustments and reinsertions; (4) rate of conversion to endotracheal intubation; (5) patient's blood pressure and heart rate at specified time points (before anesthesia induction [T0], 1 min [T1] and 2 min [T2] after induction, immediately after LMA insertion [T3], 1 min after LMA insertion [T4], 2 min after LMA insertion [T5], and 3 min after LMA insertion [T6]); (6) incidence of postoperative throat complications within 24 h; (7) visible bleeding during LMA removal; and (8) patient and instructor satisfaction.

### 2.5 Statistical analysis

As this is a pilot study and represents the first attempt of its kind in this field, there were no previously published studies or pilot data available to inform a precise effect size estimate (such as a difference in proportions or hazard ratio). Therefore, our sample size was not determined by a traditional statistical power calculation but was primarily based on clinical practicality and the study's exploratory goals. The study specifically recruited novice LMA operators. A total of 20 eligible beginners were enrolled, and all were included in the analysis. Finally, 30 patients were included in each group. The statistical analysis was performed using SPSS 25.0 software. Continuous variables are expressed as the mean ± standard deviation and were analyzed using the independent-samples *t*-test. Categorical variables are presented as percentages and were compared using the chi-square test. *P* < 0.05 was considered statistically significant.

## 3 Results

### 3.1 Baseline characteristics

Overall, 60 patients were analyzed; there were no dropouts (Figure 1). The learner–instructor ratio was 2:8 in the V-LMA group and 1:9 in the NV-LMA group, with no statistically significant difference between the two groups. Baseline demographics, including age, BMI, and sex distribution, also showed no significant differences between the two groups (Table 1).

**Table 1:** Patients' baseline characteristics.

**Patient characteristics**	**V group (*n* = 30)**	**NV group (*n* = 30)**	***P***
Age(years)	49.63 ± 16.368	53.93 ± 15.163	0.296
Male/Female	19/11	18/12	0.071
BMI(kg/m^2^)	23.373 ± 1.7815	22.777 ±1.9310	0.219

Data are expressed as the mean ± standard deviation. BMI, body mass index.

### 3.2 Primary outcome

Both groups achieved 100% first-attempt insertion success with no cases of conversion to endotracheal intubation.

### 3.3 Secondary outcomes

#### 3.3.1 Time to successful insertion and bronchoscope grade

The average time to successful insertion was 17.77 ± 7.29 s in the V-LMA group and 14.57 ± 6.79 s in the NV-LMA group, showing no statistically significant difference (*P* = 0.084; Table 2). In the V-LMA group, 90% of patients achieved bronchoscope grade 1 compared with only 50% in the NV-LMA group (*P* = 0.001). Correspondingly, fewer *in situ* adjustments were required in the V-LMA group (10% vs. 50%, *P* = 0.003).

**Table 2:** LMA insertion status in the V-LMA and NV-LMA groups.

**LMA insertion status**	**V group (*n* = 30)**	**NV group (*n* = 30)**	***P***
Time required for successful insertion (seconds)	17.77 ± 7.29	14.57 ± 6.79	0.084
Bronchoscope grade of I (*n*, %)	27 (90%)^*^	15 (50%)	0.001
*In-situ* adjustment (*n*, %)	3 (10%)^*^	15 (50%)	0.003

Data are expressed as number of patients (%). ^*^Indicates *P* < 0.05 between the two groups. LMA, laryngeal mask airway; NV-LMA, non-video laryngeal mask airway; V-LMA, video laryngeal mask airway.

#### 3.3.2 Mean arterial pressure and heart rate

**Figure 2:**
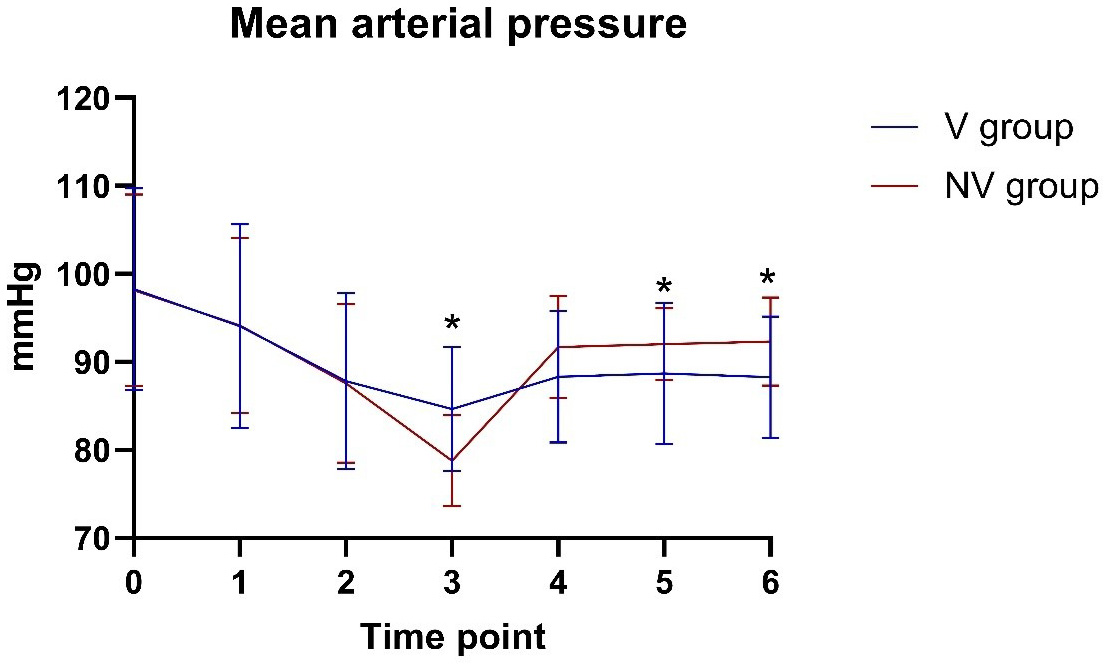
Mean arterial pressure at different time points in the V-LMA and NV-LMA groups. *P < 0.05 compared with the NV-LMA group. NV-LMA, non-video laryngeal mask airway; V-LMA, video laryngeal mask airway.

**Figure 3:**
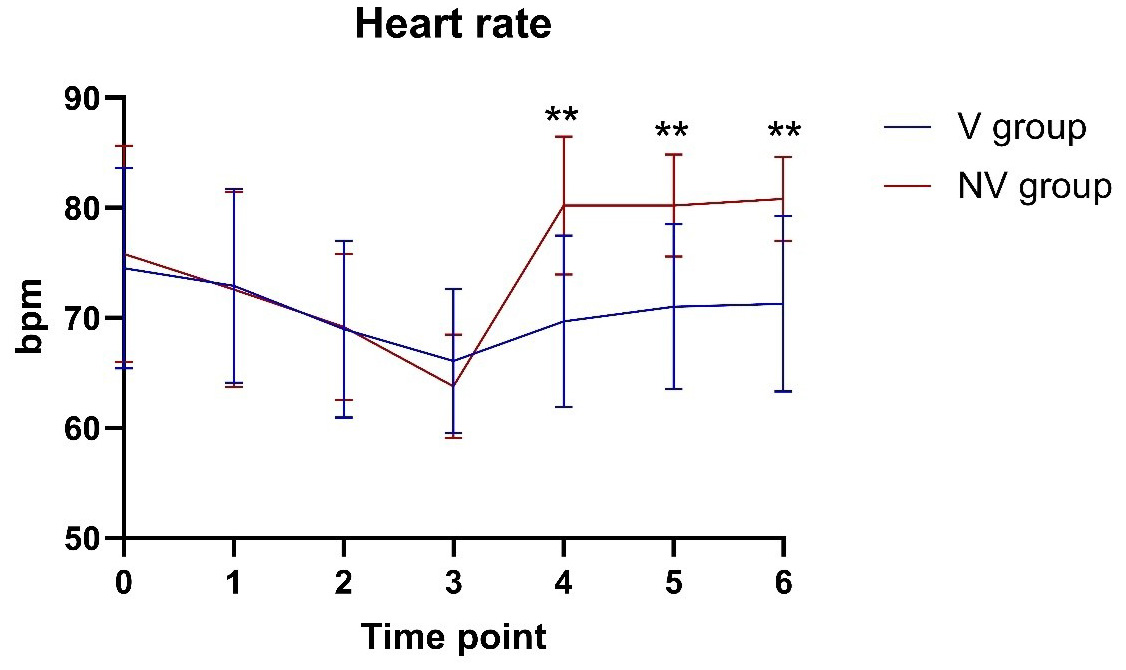
Heart rate at different time points in the V-LMA and NV-LMA groups. **P < 0.001 compared with the NV-LMA group. NV-LMA, non-video laryngeal mask airway; V-LMA, video laryngeal mask airway.

The mean arterial pressure was higher in the V-LMA group than in the NV-LMA group at T3 (84.63 ± 7.05 mmHg vs. 78.77 ± 5.17 mmHg, *P* = 0.001) (Figure 2). At T5 and T6, the mean arterial pressure was significantly lower in the V-LMA group than in the NV-LMA group (88.67 ± 8.05 mmHg vs. 92.00 ± 4.09 mmHg, *P* = 0.049, and 88.27 ± 6.88 mmHg vs. 92.33 ± 5.00 mmHg, *P* = 0.011, respectively). The V-LMA group had smaller fluctuations in mean arterial pressure than the NV-LMA group. Figure 3 shows significant differences in heart rate at different time points. At T4, T5, and T6, heart rate in the V-LMA group was significantly lower than in the NV-LMA group (T4: 69.67 ± 7.82 bpm vs. 80.20 ± 6.29 bpm, *P* < 0.001; T5: 71.03 ± 7.53 bpm vs. 80.20 ± 4.66 bpm, *P* < 0.001; T6: 71.3 ± 7.97 bpm vs. 80.83 ± 3.83 bpm, *P* < 0.001).

#### 3.3.3 Postoperative throat complications within 24 h

No severe complications (nausea, vomiting, or hoarseness) occurred within 24 h postoperatively. Blood stains on the LMA surface were observed in 20% of the patients in the V-LMA group and in 26.7% of the patients in the NV-LMA group (*P* = 0.542; Table 3). The incidence of postoperative throat pain at 1 h was significantly lower in the V-LMA group (20% vs. 46.7%, *P* = 0.028), although there was no significant difference at 24 h (56.7% vs. 63.3%, *P* = 0.598). The Visual Analog Scale (VAS) scores of patients with throat pain at 1 h postoperatively were all < 3. At 24 h postoperatively, there were three patients with VAS scores ≥3 in the V-LMA group compared with six in the NV-LMA group (*P* = 0.278).

**Table 3:** Blood staining on the LMA after removal, and throat pain at 1 h and 24 h postoperatively in the V-LMA and NV-LMA groups.

**Adverse events**	**V group (*n* = 30)**	**NV group (*n* = 30)**	***P***
Blood stains (*n*, %)	6 (20%)	8 (26.75%)	0.542
Postoperative throat pain after 1 h (*n*, %)	6 (20%)^*^	14 (46.7%)	0.028
Postoperative throat pain after 24 h (*n*, %)	17 (56.7%)	19 (63.3%)	0.598

Data are expressed as number of patients (%). ^*^Indicates *P* < 0.05 between the two groups. LMA, laryngeal mask airway; NV-LMA, non-video laryngeal mask airway; V-LMA, video laryngeal mask airway.

#### 3.3.4 Patient and instructor satisfaction

There were no cases of general dissatisfaction or dissatisfaction among the patients or the instructors in either group (Table 4). In terms of patient satisfaction, the “very satisfied” rate was 73.3% in the V-LMA group and 80% in the NV-LMA group, while the “satisfied” rate was 26.7% and 20%, respectively (*P* = 0.542). Among the instructors, the “very satisfied” rate was 66.7% in the V-LMA group and 76.7% in the NV-LMA group, while the “satisfied” rate was 33.3% and 23.3%, respectively (*P* = 0.390).

**Table 4:** Patient and instructor satisfaction.

**Subject**	**Satisfactory grading**	**V group**	**NV group**
Patient	Very satisfactory (*n*, %)	22 (73.3%)	24 (80%)
	Satisfactory (*n*, %)	8 (26.7%)	6 (20%)
	Common (*n*, %)	0 (0%)	0 (0%)
	Unsatisfactory (*n*, %)	0 (0%)	0 (0%)
Instructor	Very satisfactory (*n*, %)	20 (66.7%)	23 (76.7%)
	Satisfactory (*n*, %)	10 (33.3%)	7 (23.3%)
	Common (*n*, %)	0 (0%)	0 (0%)
	Unsatisfactory (*n*, %)	0 (0%)	0 (0%)

Data are expressed as number of patients or instructors (%).

## 4 Discussion

In recent years, the widespread application of LMAs in clinical practice, particularly in cases of difficult airway management (20–22), underscores the need to expand training beyond anesthesiologists. This study highlights the benefits of V-LMAs over traditional NV-LMAs in novice training, demonstrating higher placement accuracy, reduced complications, and enhanced hemodynamic stability among patients.

Second-generation LMAs provide numerous advantageous features; however, their optimal placement depends on accurate estimation of both LMA size and insertion depth. Anesthesiologists usually depend on a series of subjective indirect assessments and tests (23–25). According to one study, even when there are no clinical signs of air leakage, only 33% of patients have ideally positioned LMAs during blind insertion (26). Other scholars have compared blind insertion with LMA insertion under laryngoscope guidance. While the success rate of LMA insertion was close to 100% with both methods, the probability of achieving an ideal position was only 42% in the blind insertion group (27). In contrast, V-LMA insertion enables real-time visualization, allowing operators to dynamically adjust the positioning, achieving a glottic alignment rate of 94% (28). Additionally, operators can monitor the glottis and its surrounding area during the entire surgical procedure. Therefore, V-LMAs offer significant advantages in airway management.

In previous studies, the first-attempt success rate of SaCo VLM insertion was 91.4%−95%, higher than the rate of 77%−88% reported for traditional LMAs (18, 19, 29–33). In the present study, both groups achieved a first-attempt success rate of 100%. The first-attempt success rate in the V-LMA group was approximate to previous studies because the anesthesiologists were experienced in the use of SaCo VLM. In previous studies, no muscle relaxants were used before LMA insertion, which may have led to the lower first-attempt success rate than observed in our study.

The V-LMA group demonstrated significantly better bronchoscope alignment than the NV-LMA group (Grade 1: 90% vs. 50%, *P* = 0.001), consistent with a previous trial showing 91.4% accuracy for SaCo VLM vs. LMA Supreme (29). In previous studies, 40%−60% of the blindly intubated laryngeal masks did not achieve perfect positioning with a broncho-fiberscope, requiring realignment to improve ventilation (26, 34).

The reported incidence of postoperative throat pain can be as high as 70.6% (35–39). In the present study, the incidence of throat pain 1 h after the procedure was 20%, while it was 56.7% at 24 h after the procedure, markedly lower than reported previously. Potential causes of throat pain include deep LMA placement, epiglottis folding, violent blind insertion owing to poor visualization of the oral cavity, LMA material, and LMA oversizing. In the present study, patients in both groups experienced varying degrees of throat pain postoperatively, but the incidence was lower than reported in the literature, and the NV-LMA group had a higher rate than the V-LMA group. This may be related to the use of non-steroidal anti-inflammatory drugs during surgery, instructor guidance during LMA placement, controlled LMA pressure during surgery, and timely improvements in cases of misalignment or deep placement.

Two studies on NV-LMAs have reported blood stains on the LMA after removal in 7%−10% of patients (40, 41). In the present study, the incidence of blood stains on the LMA surface after removal was 26.75% in the NV-LMA group and 20% in the V-LMA group, higher than in previous studies. This may be related to the inexperience of the novice operators, insufficiently gentle LMA removal, or the LMA material. The lower incidence in the V-LMA group may be due to the operators having a better sense of direction during insertion under direct visualization.

Although the insertion time tended to be longer in the V-LMA group, there was no statistically significant difference when compared with the NV-LMA group. This is logical as novices in the V-LMA group took more time to assess the oral cavity structure and insertion path during the learning phase than those in the NV-LMA group who relied on blind insertion based on experience.

Hemodynamic analysis during LMA insertion showed that changes in patient hemodynamics after LMA insertion were within 20% of baseline values in both groups, indicating that the LMA is a safe and reliable method for airway management in terms of maintaining hemodynamic stability. Additionally, despite the longer insertion time in the V-LMA group, mean arterial pressure and heart rate were more stable before and after insertion. Direct visualization likely mitigated excessive tissue manipulation, reducing sympathetic stimulation.

The high rates of satisfaction among patients and instructors in both groups aligns with the low complication rates in this study, and there were no statistically significant differences in satisfaction between the two groups.

### 4.1 Limitations

This study had several limitations that should be considered when interpreting the findings. First, this was a single-center study with a small sample size. Therefore, large multicenter studies will be needed in the future to validate the findings. Second, LMA size was determined according to the patient's weight, without accounting for sex-specific anatomical variations (42). Third, the absence of initial learning-phase metrics (e.g., early failure rates) on manikins during learning phase limited a comprehensive evaluation of skill acquisition. Forth, this study was designed as a pilot and not powered to detect statistically significant differences between groups. Finally, as the novices were not blinded, potential assessment bias may exist. In the future, large multicenter studies are needed to validate the learning outcomes of V-LMA.

## 5 Conclusion

In summary, the V-LMA enhanced placement accuracy through real-time visualization, enabling immediate adjustments and reducing complications, such as postoperative throat pain and hemodynamic instability. These advantages make the V-LMA particularly suitable for novice practitioners and improve patient safety.

## Data Availability

The original contributions presented in the study are included in the article/supplementary material, further inquiries can be directed to the corresponding authors.

## References

[1] Jannu A Shekar A Balakrishna R Sudarshan H Veena GC Bhuvaneshwari S. Advantages, disadvantages, indications, contraindications and surgical technique of laryngeal airway mask. Arch Craniofac Surg. (2017) 18:223–9. 10.7181/acfs.2017.18.4.223 29349045 PMC5759658

[2] Brain AI. The laryngeal mask–a new concept in airway management. Br J Anaesth. (1983) 55:801–5. 10.1093/bja/55.8.801 6349667

[3] Ramesh S Jayanthi R. Supraglottic airway devices in children. Indian J Anaesth. (2011) 55:476–82. 10.4103/0019-5049.89874 22174464 PMC3237147

[4] Bein B Scholz J. Supraglottic airway devices. Best Pract Res Clin Anaesthesiol. (2005) 19:581–93. 10.1016/j.bpa.2005.08.005 16408535

[5] Van Zundert AAJ Kumar CM Van Zundert T Gatt SP Pandit JJ. The case for a 3rd generation supraglottic airway device facilitating direct vision placement. J Clin Monit Comput. (2021) 35:217–24. 10.1007/s10877-020-00537-4 32537697 PMC7293959

[6] Heidegger T Gerig HJ. Algorithms for management of the difficult airway. Curr Opin Anaesthesiol. (2004) 17:483–4. 10.1097/00001503-200412000-00004 17031079

[7] Schäuble JC Heidegger T. Management of the difficult airway: overview of the current guidelines. Anaesthesist. (2018) 67:725–37. 10.1007/s00101-018-0492-8 30291405

[8] Furman WR. Hagberg and Benumof's Airway Management. 4th ed, New York: Elsevier Health Sciences. (2018). 10.1213/ANE.0000000000003475

[9] Candido KD Saatee S Appavu SK Khorasani A. Revisiting the ASA guidelines for management of a difficult airway. Anesthesiology. (2000) 93:295–8. 10.1097/00000542-200007000-00050 10861180

[10] Yan CL Zhang YQ Chen Y Qv ZY Zuo MZ. Comparison of SaCoVLM™ video laryngeal mask-guided intubation and i-gel combined with flexible bronchoscopy-guided intubation in airway management during general anesthesia: a non-inferiority study. BMC Anesthesiol. (2022) 22:302. 10.1186/s12871-022-01843-x 36138363 PMC9494909

[11] Fang F Jin J Pi Y Guo S Li Y Zhu S. Emergency endotracheal intubation in critically ill patients with COVID-19: management and clinical characteristics. Anesthesiol Perioperat Sci. (2023) 1:7. 10.1007/s44254-023-00003-9 40476902 PMC10008717

[12] Van Zundert AAJ Gatt SP Kumar CM Van Zundert T Pandit JJ. ‘Failed supraglottic airway': an algorithm for suboptimally placed supraglottic airway devices based on videolaryngoscopy. Br J Anaesth. (2017) 118:645–9. 10.1093/bja/aex093 28510747

[13] Van Zundert AAJ Gatt SP Van Zundert T Kumar CM Pandit JJ. Features of new vision-incorporated third-generation video laryngeal mask airways. J Clin Monit Comput. (2022) 36:921–8. 10.1007/s10877-021-00780-3 34919170

[14] Brimacombe JR. Problems with the laryngeal mask airway: prevention and management. Int Anesthesiol Clin. (1998) 36:139–54. 10.1097/00004311-199803620-00011 9704277

[15] Brimacombe JR Berry AM White PF. The laryngeal mask airway: limitations and controversies. Int Anesthesiol Clin. (1998) 36:155–82. 10.1097/00004311-199803620-00012 9704278

[16] Liu Y He Y Wang X Li J Zhang Z Zhuang X. Advances in airway management in recent 10 years from 2013 to 2023. Anesthesiol Perioperat Sci. (2023) 1:27. 10.1007/s44254-023-00029-z

[17] Brimacombe J Berry A. A proposed fiber-optic scoring system to standardize the assessment of laryngeal mask airway position. Anesth Analg. (1993) 76:457. 8424538

[18] Kim GW Kim JY Kim SJ Moon YR Park EJ Park SY. Conditions for laryngeal mask airway placement in terms of oropharyngeal leak pressure: a comparison between blind insertion and laryngoscope-guided insertion. BMC Anesthesiol. (2019) 19:4. 10.1186/s12871-018-0674-6 30611202 PMC6320569

[19] Yan CL Chen Y Sun P Qv ZY Zuo MZ. Preliminary evaluation of SaCoVLM™ video laryngeal mask airway in airway management for general anesthesia. BMC Anesthesiol. (2022) 22:3. 10.1186/s12871-021-01541-0 34979936 PMC8722220

[20] Higgs A McGrath BA Goddard C Rangasami J Suntharalingam G Gale R. Guidelines for the management of tracheal intubation in critically ill adults. Br J Anaesth. (2018) 120:323–52. 10.1016/j.bja.2017.10.021 29406182

[21] Frerk C Mitchell VS McNarry AF Mendonca C Bhagrath R Patel A. Difficult Airway Society 2015 guidelines for management of unanticipated difficult intubation in adults. Br J Anaesth. (2015) 115:827–48. 10.1093/bja/aev371 26556848 PMC4650961

[22] Batuwitage B Charters P. Postoperative management of the difficult airway. BJA Educ. (2017) 17:235–41. 10.1093/bjaed/mkw077

[23] Christodoulou C. ProSeal laryngeal mask airway foldover detection. Anesth Analg. (2004) 99:312–3. 10.1213/01.ANE.0000127714.32845.7F 15281564

[24] O'Connor Jr CJ Borromeo CJ Stix MS. Assessing ProSeal laryngeal mask positioning: the suprasternal notch test. Anesth Analg. (2002) 94:1374–5. 10.1097/00000539-200205000-00082 11973233

[25] Hartopp R Maynard JP. Failed gastric tube insertion in the LMA-ProSeal. Anaesthesia. (2004) 59:827. 10.1111/j.1365-2044.2004.03881.x 15300953

[26] Campbell RL Biddle C Assudmi N Campbell JR Hotchkiss M. Fiberoptic assessment of laryngeal mask airway placement: blind insertion versus direct visual epiglottoscopy. J Oral Maxillofac Surg. (2004) 62:1108–13. 10.1016/j.joms.2003.10.014 15346362

[27] Van Zundert AA Kumar CM Van Zundert TC. Malpositioning of supraglottic airway devices: preventive and corrective strategies. Br J Anaesth. (2016) 116:579–82. 10.1093/bja/aew104 27106958

[28] van Zundert AAJ Wyssusek KH Pelecanos A Roets M Kumar CM. A prospective randomized comparison of airway seal using the novel vision-guided insertion of LMA-Supreme^®^ and LMA-Protector^®^. J Clin Monit Comput. (2020) 34:285–94. 10.1007/s10877-019-00301-3 30953222

[29] Sun Y Zhang M Gao X Gao Z Zou T Guo Y. Effect of the new video laryngeal mask airway SaCoVLM on airway management in lateral laparoscopic urological surgery: a single center randomized controlled trial. Sci Rep. (2024) 14:2132. 10.1038/s41598-024-54129-2 38272937 PMC10810894

[30] Eschertzhuber S Brimacombe J Hohlrieder M Keller C. The laryngeal mask airway Supreme–a single use laryngeal mask airway with an oesophageal vent. A randomised, cross-over study with the laryngeal mask airway ProSeal in paralysed, anaesthetised patients. Anaesthesia. (2009) 64:79–83. 10.1111/j.1365-2044.2008.05682.x 19087011

[31] Mukadder S Zekine B Erdogan KG Ulku O Muharrem U Saim Y. Comparison of the proseal, supreme, and i-gel SAD in gynecological laparoscopic surgeries. ScientificWorldJournal. (2015) 2015:634320. 10.1155/2015/634320 25802890 PMC4353657

[32] Kriege M Piepho T Zanker S Alflen C Heid F Noppens RR. LMA Supreme™ and Ambu^®^ AuraGain™ in anesthetized adult patients: a prospective observational study. Minerva Anestesiol. (2017) 83:165–74. 10.23736/S0375-9393.16.11112-5 27676414

[33] Chaw SH Shariffuddin II Foo LL Lee PK Paran RM Cheang PC. Comparison of clinical performance of size 1.5 Supreme™ LMA and Proseal™ LMA among Asian children: a randomized controlled trial. J Clin Monit Comput. (2018) 32:1093–1099. 10.1007/s10877-018-0109-4 29404890

[34] Chandan SN Sharma SM Raveendra US Rajendra Prasad B. Fiberoptic assessment of laryngeal mask airway placement: a comparison of blind insertion and insertion with the use of a laryngoscope. J Maxillofac Oral Surg. (2009) 8:95–8. 10.1007/s12663-009-0025-8 23139483 PMC3453942

[35] Kömür E Bakan N Tomruk G Karaören SG Dogan ZT. Comparison of the supraglottic airway devices classic, fastrach and supreme laryngeal mask airway: a prospective randomised clinical trial of efficacy, safety and complications. Turk J Anaesthesiol Reanim. (2015) 43:406–11. 10.5152/TJAR.2015.97830 27366537 PMC4894184

[36] Tan BH Chen EG Liu EH. An evaluation of the laryngeal mask airway supreme' in 100 patients. Anaesth Intensive Care. (2010) 38:550–4. 10.1177/0310057X1003800322 20514967

[37] López AM Valero R Brimacombe J. Insertion and use of the LMA Supreme in the prone position. Anaesthesia. (2010) 65:154–7. 10.1111/j.1365-2044.2009.06185.x 19930218

[38] Evans NR Gardner SV James MF King JA Roux P Bennett P. The proseal laryngeal mask: results of a descriptive trial with experience of 300 cases. Br J Anaesth. (2002) 88:534–9. 10.1093/bja/88.4.534 12066730

[39] Seet E Rajeev S Firoz T Yousaf F Wong J Wong DT. Safety and efficacy of laryngeal mask airway Supreme versus laryngeal mask airway ProSeal: a randomized controlled trial. Eur J Anaesthesiol. (2010) 27:602–7. 10.1097/EJA.0b013e32833679e3 20540172

[40] Anand LK Goel N Singh M Kapoor D. Comparison of the Supreme and the ProSeal laryngeal mask airway in patients undergoing laparoscopic cholecystectomy: a randomized controlled trial. Acta Anaesthesiol Taiwan. (2016) 54:44–50. 10.1016/j.aat.2016.03.001 27106162

[41] Wender R Goldman AJ. Awake insertion of the fibreoptic intubating LMA CTrach in three morbidly obese patients with potentially difficult airways. Anaesthesia. (2007) 62:948–51. 10.1111/j.1365-2044.2007.05127.x 17697225

[42] Kihara S Brimacombe JR Yaguchi Y Taguchi N Watanabe S. A comparison of sex- and weight-based ProSeal laryngeal mask size selection criteria: a randomized study of healthy anesthetized, paralyzed adult patients. Anesthesiology. (2004) 101:340–3. 10.1097/00000542-200408000-00014 15277916

